# Nanostructured liquid crystal systems and applications

**DOI:** 10.3762/bjnano.9.245

**Published:** 2018-10-05

**Authors:** Alexei R Khokhlov, Alexander V Emelyanenko

**Affiliations:** 1 Lomonosov Moscow State University, Faculty of Physics, Leninskie Gory, Moscow 119991, Russia

**Keywords:** artificial muscles, displays, energy saving, liquid crystals, photoinduction, sensors

Liquid crystals are smart materials having numerous applications in liquid crystal displays, modulators, sensors, solar cells, etc. Liquid crystals are used in both large and small devices. They surround us everywhere: in our house, on the street, and at our job. Liquid crystals are applied in biology and medicine, and for oil recovery as well as in food production. The molecules of the human body (e.g., DNA, proteins) can also form liquid crystal phases. Many applications of liquid crystals require the manipulation of structures on the nanometer scale. For example, these highly sensitive materials are capable of changing their structure on the nanoscale level for detection of small fractions of dopant materials. In this respect, much attention has been paid to the investigation of liquid crystal droplets dissolved in liquid, in particular, to the photoinduced orientation of liquid crystal molecules at the surface of a droplet. There have also been reports of some very interesting experiments with nanocomposites where the solid nanoparticles are dissolved in liquid crystals. Composite materials and mixtures of liquid crystals usually possess better properties than their pure liquid crystal counterparts. Many challenges could be potentially solved with help of liquid crystals, such as the creation of artificial muscles and global energy reduction.

Liquid crystals were discovered in 1888 by the Austrian botanist Friedrich Reinitzer [1–3] who observed the “two liquid states” in cholesteryl benzoate. However, for many years, no practical interest in liquid crystals was found. Much later, in 1927, the effect of the orientation of liquid crystals by an electric field was discovered by Russian physicist Vsevolod Fréedericksz [4–6]. Today, the operation of the liquid crystal display (LCD) is based on this effect named after him. However, this effect did not find application at that time. It was not until 1965 that the active investigation of liquid crystals began, and the first display device using the twisted nematic field was patented by the American inventor James Fergason in 1969. LCDs are now an industry that generates over ten billion dollars per year.

In the 21st century, the topic of energy consumption has become a topic of critical concern for the future survival of the next generations. The new energy-saving liquid crystal devices are therefore of prime importance in industry. This in turn indicates that investigations of liquid crystals on the nanoscale are essential for the survival and development of civilization. Most everyone today in the modern world uses multiple display devices (e.g., TV, computer monitors, smartphones, projectors). Most of such displays are based on nematic liquid crystals, in which each pixel consists of the three sub-pixels (red, green and blue), and thus, up to 2/3 of the backlight can be absorbed by the color filters. Although the energy consumption by each display does not appear to be very large, with the whole human population using displays, this results in a huge waste of energy, equivalent to that generated by several nuclear power stations. Many scientific groups all over the world are engaged in the development of new energy-saving display materials. One of the newest ideas is to create a field sequential color liquid crystal display [7], in which each pixel is synchronized with the operation of a light matrix in a way that red, green and blue colors propagate through the same pixel during a particular time. In this way, the human eye mixes all the colors in a particular proportion to obtain an arbitrary color. The elaboration of the field sequential color displays should result in much cheaper devices, while their color gamut, brightness and resolution will also be much better. The new displays will also save up to 70% of energy. For this purpose, the several times “faster” liquid crystals are required than are used now.

Finally, liquid crystals are simply fantastic and beautiful materials. The investigation of liquid crystals makes an important contribution to the development of new scientific methods and approaches in mathematics, computer science, molecular-statistical physics, thermodynamics, electrodynamics, etc. The methods derived in the framework for the research of liquid crystals could be applied in different areas, and therefore, liquid crystal research is intended to stimulate progress in science in general.

This thematic issue is devoted to nanostructured liquid crystal (LC) systems and applications. It includes several directions, such as

- novel LC phases, structure and phase behavior
- design and synthesis of LC materials
- photonic, electro- and photo-responsive LC systems
- theory and simulations of LC systems
- LC polymers, elastomers, colloids and gels
- hybrid and nanostructured LC systems
- biological, lyotropic and chromonic LC systems
- ferroelectric LC systems
- confined LC systems and defects
- hydrodynamics and microfluidics of LC systems
- applications of liquid crystals

We believe that this thematic issue will be of interest to many readers working in different fields of research, including interdisciplinary research. We are grateful to the participants of the 14th European Conference on Liquid Crystals (Moscow, 2017) [8], many of whom contributed to this issue.

Alexander Emelyanenko and Alexei Khokhlov

Moscow, September 2018
